# Isovaline Does Not Activate GABA_B_ Receptor-Coupled Potassium Currents in GABA_B_ Expressing AtT-20 Cells and Cultured Rat Hippocampal Neurons

**DOI:** 10.1371/journal.pone.0118497

**Published:** 2015-02-23

**Authors:** Kimberley A. Pitman, Stephanie L. Borgland, Bernard MacLeod, Ernest Puil

**Affiliations:** 1 Department of Anesthesiology, Pharmacology & Therapeutics, University of British Columbia, Vancouver, British Columbia, Canada; 2 Hotchkiss Brain Institute, University of Calgary, Calgary, Alberta, Canada; Dalhousie University, CANADA

## Abstract

Isovaline is a non-proteinogenic amino acid that has analgesic properties. R-isovaline is a proposed agonist of the γ-aminobutyric acid type B (GABA_B_) receptor in the thalamus and peripheral tissue. Interestingly, the responses to R-isovaline differ from those of the canonical GABA_B_ receptor agonist R-baclofen, warranting further investigation. Using whole cell recording techniques we explored isovaline actions on GABA_B_ receptors coupled to rectifying K^+^ channels in cells of recombinant and native receptor preparations. In AtT-20 cells transfected with GABA_B_ receptor subunits, bath application of the GABA_B_ receptor agonists, GABA (1 μM) and R-baclofen (5 μM) produced inwardly rectifying currents that reversed approximately at the calculated reversal potential for K^+^ R- isovaline (50 μM to 1 mM) and S-isovaline (500 μM) did not evoke a current. R-isovaline applied either extracellularly (250 μM) or intracellularly (10 μM) did not alter responses to GABA at 1 μM. Co-administration of R-isovaline (250 μM) with a low concentration (10 nM) of GABA did not result in a response. In cultured rat hippocampal neurons that natively express GABA_B_ receptors, R-baclofen (5 μM) induced GABA_B_ receptor-dependent inward currents. Under the same conditions R-isovaline (1 or 50 μM) did not evoke a current or significantly alter R-baclofen-induced effects. Therefore, R-isovaline does not interact with recombinant or native GABA_B_ receptors to open K^+^ channels in these preparations.

## Introduction

Receptors for γ-aminobutyric acid (GABA), especially of the GABA_B_ type, are a promising target in analgesia. However, the use of the prototypical GABA_B_ agonist, baclofen, is limited due to the development of severe side effects [1]. Isovaline is an unusual non-proteinogenic amino acid that is anti-nociceptive without side effects typical of GABA_B_ agonists [2,3]. A component of the analgesia produced by isovaline is attributable to activation of GABA_B_ receptors [2].

GABA_B_ receptors are obligate heterodimers, made up of a GABA_B1_ and a GABA_B2_ subunit [4–6]. Surprisingly, there are no pharmacologically distinct GABA_B_ receptor subtypes [7,8]. Critical residues for orthosteric agonist and antagonist binding are located on the GABA_B1_ subunit within a region known as the Venus flytrap domain [9–11], while allosteric modulators of the GABA_B_ receptor act at sites on the GABA_B2_ subunit [12]. GABA_B_ receptors are G-protein coupled receptors that associate with the pertussis toxin sensitive G_αi/o_ family of G-proteins [13]. Conventional cellular effects of GABA_B_ receptor agonism include inhibition of adenylate cyclase, G_βγ_-mediated activation of G-protein coupled inwardly rectifying K^+^ (GIRK) channels and G_βγ_-mediated inhibition of voltage gated Ca^2+^ channels [8,14,15].

Isovaline’s action at GABA_B_ receptors is atypical as demonstrated in thalamic neurons of brain slices. Compared to R-baclofen, R-isovaline-evoked K^+^ currents are slow in onset and long lasting [16,17]. Also in contrast to R-baclofen, the response to R-isovaline is blocked by pre-application of a GABA_B_ receptor antagonist, but not by application of the GABA_B_ antagonist subsequent to the initiation of the K^+^ current [16]. Furthermore, some neurons that respond to R-baclofen do not respond to R-isovaline [16]. The ability of R-isovaline to activate currents in sensitive neurons does not appear to result from GABA release and subsequent postsynaptic activation of GABA_B_-mediated K^+^ currents, since inhibition of other GABA-mediated currents does not alter R-isovaline responses [16]. Here we test the effects of R-isovaline on GABA_B_ receptors in a neuronal expression system as well as in isolated hippocampal neurons natively expressing GABA_B_ receptors.

## Materials and Methods

### AtT-20 cell culturing, transfection and electrophysiology

Mouse pituitary AtT-20 cells [18,19] obtained from Dr. C. Chavkin’s laboratory at the University of Washington (March, 2010) were grown in Gibco high glucose Dulbecco’s modified Eagle medium (Invitrogen) with 1% fetal bovine Serum, 10% horse serum, penicillin-streptomycin and 0.2 mM L-glutamine and kept in an incubator at 37°C with 5% CO_2_. Cells were passaged every 2–3 days. For transient transfection cells were plated in a 35mm culture dish at 90% confluency and left for 24 hours. Cells were then co-transfected overnight in serum containing media using Lipofectamine 2000 (Invitrogen, Life technologies, Burlington, ON) following the manufacturer’s protocols. Cells were co-transfected with cDNA for the GABA_B1a_ and GABA_B2_ subunits as well as green fluorescent protein (GFP) to aid identification of transfected cells (1:2:5 ratio of GFP:GABA_B1a_:GABA_B2_). The following day the transfection media were removed and cells were re-plated onto poly-D-lysine coated glass cover slips which were then incubated for a further 24 hours before use.

To confirm membrane expression of the GABA_B_ receptor in cells that were suitable for patching (isolated from one another), we performed immunohistochemistry for the GABA_B1_ subunit which is unable to traffic to the membrane without associating with a GABA_B2_ subunit [4–6]. Cells were fixed with 4% formaldehyde for 30 minutes then blocked with 0.5% bovine serum albumin for 1 hour. Cells were incubated with or without mouse anti-GABA_B1_ (1:1000 dilution, ab55051, Abcam, Toronto) overnight at 4°C. Goat anti-mouse conjugated to Alexa Fluor 546 (Life technologies) was applied at a 1:200 dilution for 1 hour at room temperature. DAPI (4',6-diamidino-2-phenylindole) was applied at 1:10,000 dilution for 5 minutes to stain the nuclei. Coverslips were washed with phosphate buffered saline 3 times between each step. The glass coverslips were mounted onto glass slides and left to dry for at least 72 hours. Confocal microscopy was performed using an Olympus FluoView FV1000 confocal microscope with a 60x objective (Tokyo, Japan).

For electrophysiological recordings, cells were removed from the incubator and the medium was replaced at room temperature with high K^+^ extracellular solution containing (in mM): NaCl (130), KCL (20), CaCl_2_ (2), MgCl_2_ (1), HEPES (10), glucose (35), and 0.1–1 μM tetrodotoxin. The solution had a pH = 7.4 (adjusted with NaOH) and an osmolality of 330 ± 5 mOsm. The glass coverslip was cut into sections and placed into a 50 μL fast exchange diamond bath (Warner Instruments, Hamden, CT) constantly perfused (∼2 ml/min) with extracellular solution. Recording electrodes were made out of thin-walled borosilicate glass (World Precision Instruments, Sarasota, FL) and had a resistance between 3–5 MΩ when filled with an intracellular pipette solution containing (in mM): K-gluconate (130), NaCl (20), MgCl_2_ (1), EGTA (10), glucose (10), HEPES (10), MgATP (5) and Na_3_GTP (0.1) with pH = 7.2 (NaOH/HCl) and an osmolality of 310 ± 3 mOsm. The calculated Nernst equilibrium potential for K^+^ was approximately -47 mV. Cells were visualized with an Axiovert 25 inverted fluorescent microscope (Zeiss, Germany). Whole cell recordings from fluorescing cells were performed using a List EPC 7 amplifier (HEKA, Germany). Recordings were filtered at 3 kHz, digitized at 10 kHz and analysed using pClamp 8.2 software (Molecular Devices, Sunnyvale, CA). Cells were voltage-clamped at -50 mV. The current-voltage (IV) relationship was measured each minute by applying a family of 400 ms voltage steps, from -100 mV to +20 mV, in 10 mV increments. A junction potential of -12.5 mV was accounted for offline. The current was measured as the average current in the last 100 ms of each voltage step. In all experiments except one, drugs were applied via the bath for 2–5 min, and then washed off. In one experiment R-isovaline was added into the intracellular pipette solution. Cells were held in voltage clamp for an extra 5 min before further drug application to allow for intracellular dialysis. In several cells GABA_B_ action was confirmed by blockade with a GABA_B_ antagonist.

### Hippocampal cell culturing and electrophysiology

Cultured hippocampal neurons were prepared from 18 day old Sprague-Dawley rat embryos (male and female) following methods described in detail by Xie et al. [20]. Rat housing and culture preparation methods were in accordance with the Canadian Council on Animal Care and approved by the University of British Columbia Animal Care Committee. Pregnant rats were sacrificed with 5% CO_2_ and care was taken to ensure an absence of nociceptive response prior to decapitation and removal of embryos. Neurons were plated on poly-L-lysine coated glass coverslips at a density of 130 cells/mm^2^ and were incubated with 5% CO_2_ at 37°C for 14 to 28 days until used for electrophysiological analysis *in vitro*.

On the day of recording cultured hippocampal neurons were removed from the incubator and the glass coverslip was placed into a culture dish filled with room temperature (∼22°C) extracellular solution containing (in mM): NaCl (140), KCl (5.4), CaCl_2_ (2), MgCl_2_ (1), HEPES (20) and glucose (20) at pH = 7.4 and 325 ± 5 mOsm. In some experiments, hippocampal neurons were incubated with R-isovaline (50 μM) in extracellular solution for at least an hour before recordings. Recording electrodes (4–5.5 MΩ) were filled with a solution containing (in mM): K-gluconate (120), KCl (20), NaCl (10), HEPES (10), EGTA (5) MgATP (3) and Na_2_GTP (0.2) at pH 7.2 and 305 ± 5 mOsm. Cells were voltage clamped at -80 mV and the membrane current was constantly recorded. To enhance inward K^+^ currents, a high [K^+^] extracellular solution, whereby 20 mM NaCl was replaced with equimolar KCl, was used to examine drug effects. Drugs were mixed with the high [K^+^] extracellular solution and applied via the bath perfusate for a minimum of 30 s and a maximum of 5 min. The average current during 20 s of the peak drug effect was measured and compared with the current in the last 20 s before the drug was washed in (baseline). In the case of no obvious drug effect, the average current within the last 20 s before drug wash-off was measured. Recordings were filtered at 3 kHz, digitized at 10 kHz and analysed using pClamp 8.2 software.

### Drugs

Extracellular solution was prepared on the day of the experiment. Intracellular solution was prepared and frozen in aliquots and used within a week. Stock solutions of R-baclofen HCl (25 mM, Sigma-Aldrich, St. Louis, MO), GABA (50 mM, Sigma-Aldrich), CGP 52432 (10 mM, Tocris, UK) and R-isovaline HCl or S-isovaline HCl (100 mM, BioFine International, Vancouver, BC), were made using double distilled H_2_0 and kept at 4°C. Final drug concentrations were prepared on the day of experiment.

### Analysis and Statistics

For data from AtT-20 cells, agonist action at the GABA_B_ receptor was defined as the ability to induce a current that was inwardly rectifying and reversed at approximately E_K_. The net current was determined by subtracting the baseline current from the current during drug application at each voltage step. The IV relationships were then plotted to determine whether the net current was inwardly rectifying. To determine the reversal potential, the linear section of the baseline subtracted IV curve (-112.5 mV to -52.5 mV) for each recording was fitted using linear regression, the point at which Y = 0 for each curve was determined and mean ± SEM and 95% confidence interval calculated. For clarity, example currents from AtT-20 cells depict only the current recorded at the maximum hyperpolarising step (-112.5 mV). Bar graphs display the untransformed current at the maximum hyperpolarising step (-112.5 mV) for AtT-20 cells or at the holding potential (-80 mV) for hippocampal cells. For analysis of the effect of intracellular R-isovaline, the baseline current was measured by stepping to -112.5 mV from a holding potential of -62.5 mV at t = 1 min. This baseline value was then subtracted from all subsequent measurements. Repeated measures 1 or 2-way ANOVAs with Bonferroni’s post-hoc test were used as appropriate to test for drug induced changes in currents. For analysis of changes in the R-baclofen-evoked current in hippocampal cells, the baclofen-evoked current (net current) was determined by subtracting the baseline current from the current during application of R-baclofen. A 1-way ANOVA was used to compare the magnitude of R-baclofen-evoked currents. Data are expressed as mean ± SEM. “n” refers to the number of cells. Results were considered significant if p < 0.05.

## Results

**Fig 1:**
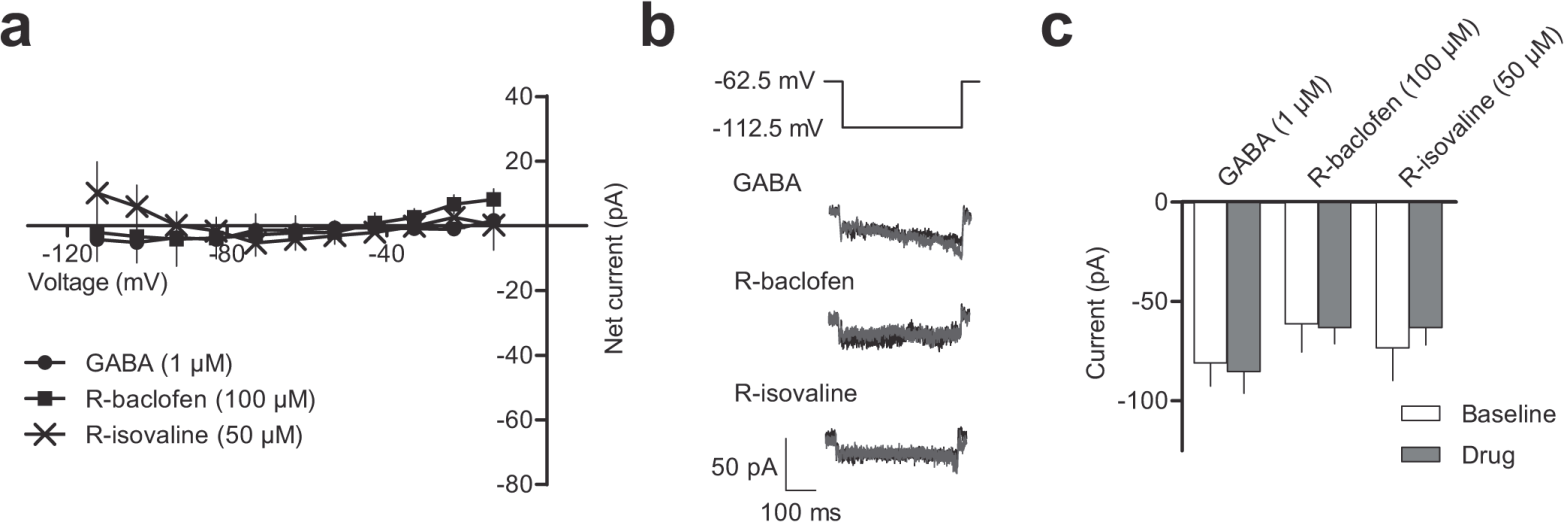
GABA, R-baclofen and R-isovaline do not evoke currents in untransfected AtT-20 cells. **a)** Baseline subtracted IV curve for GABA (n = 4), R-baclofen (n = 4) and R-isovaline (n = 5). **b**) Example currents at the maximum hyperpolarising step (-112.5 mV, voltage step depicted above currents) at baseline (black) and on application (grey) of GABA, R-baclofen or R-isovaline. **c**) Summary graph showing currents recorded at -112.5 mV at baseline (open bars) and on application of GABA, R-baclofen, or R-isovaline (shaded bars). Data are represented as mean ± SEM.

We examined whether untransfected AtT-20 cells exhibited plasmalemmal responses to applications of GABA_B_ agonists or R-isovaline by determining their effects at various membrane voltages. Fig. 1A-C shows that there was no statistically significant effects of GABA, R-baclofen or R-isovaline on the IV-relationships or on the maximal currents evoked with a hyperpolarising step to -112.5 mV (main drug effect F_(1,10)_ = 0.07, p = 0.8; repeated measures 2-way ANOVA). Bonferroni’s post hoc test indicated no changes in the current after bath application of GABA (baseline: -80.9 ± 11.5 pA vs. GABA: -85.2 ± 10.9 pA, 1 μM, 2 min, n = 4), R-baclofen (baseline: -61.2 ± 14.2 pA vs. R-baclofen: -63.2 ± 8.2 pA, 100 μM, 2 min, n = 4) or R-isovaline (baseline: -73.3 ± 16.3 pA vs. R-isovaline: -63.2 ± 8.6 pA, 50 μM, 5 min, n = 5). These data, summarized in Fig. 1C, suggest that untransfected AtT-20 cells do not have GABA_B_ receptors which couple to K^+^ currents.

**Fig 2:**
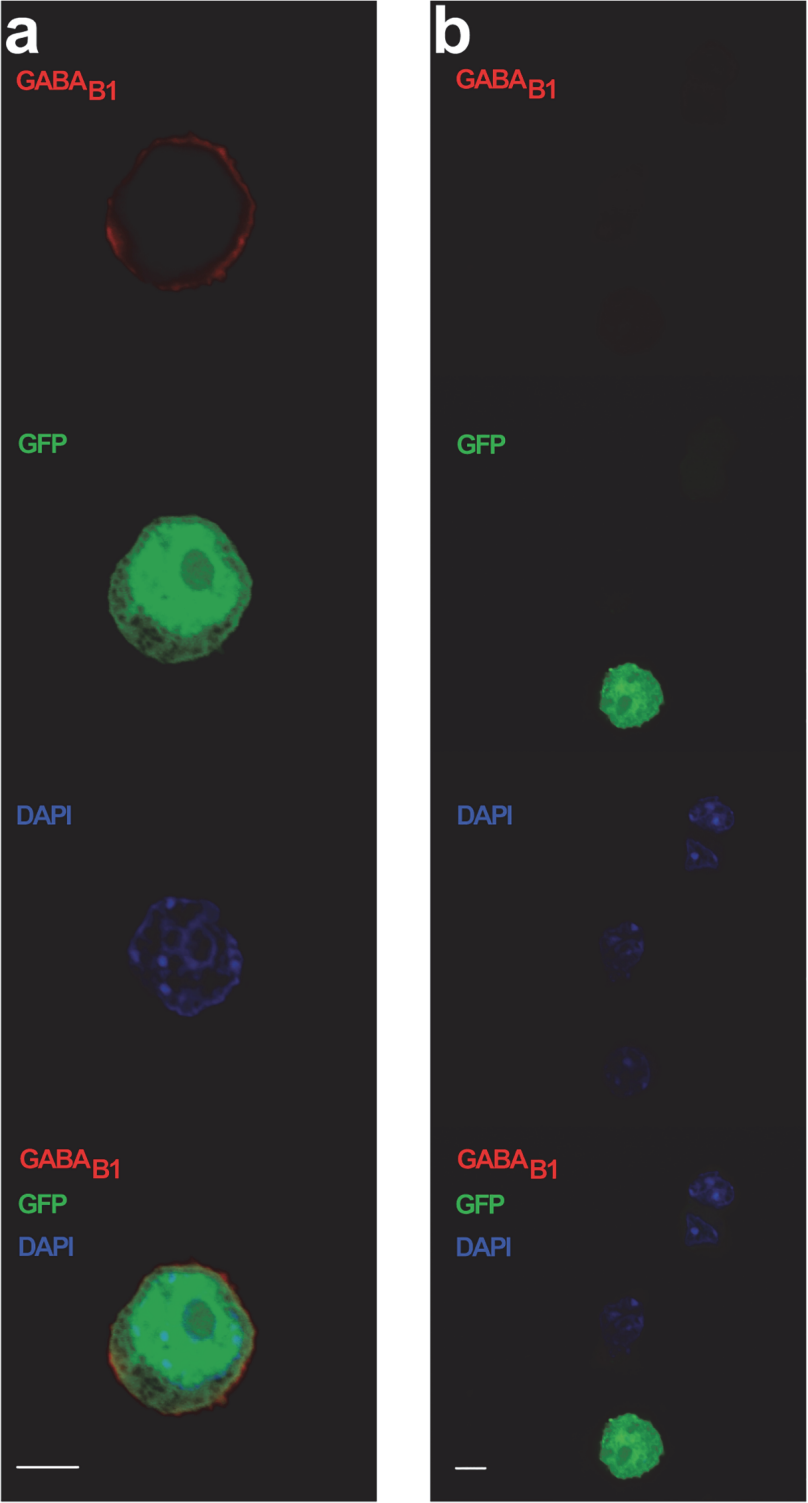
The GABA_B1_ subunit expresses at the membrane in transfected AtT-20 cells. AtT-20 cells were transfected with both subunits for the GABA_B_ receptor along with GFP (shown in green). Immunohistochemistry was performed for the GABA_B1_ subunit (red) and cell nuclei were stained with DAPI (blue). **a)** Magnified image of an isolated cell demonstrating GABA_B1_ subunit protein at the cell membrane in a GFP positive cell. **b)** Negative control (no primary antibody for GABA_B1_). A merged image is shown at the bottom of each column. Scale bars are 5 μm.

**Fig 3:**
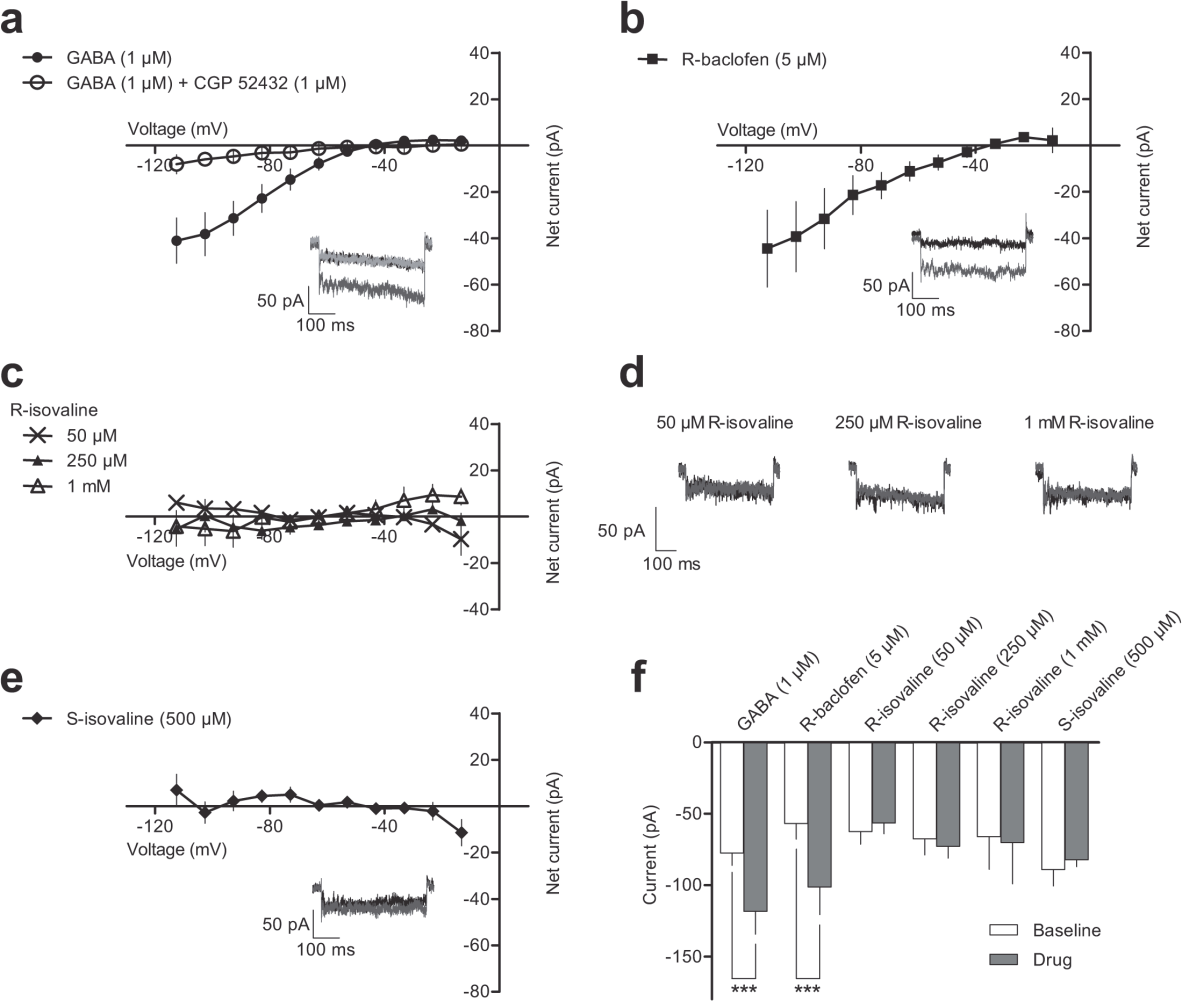
GABA and R-baclofen, but not isovaline, evoke inwardly rectifying currents in transfected AtT-20 cells. **a)** Baseline subtracted IV curve for GABA and GABA + CGP 52432 (n = 7). Inset shows example currents at baseline (black), in the presence of GABA (dark grey) or in the presence of GABA + CGP 52432 (light grey). **b**) Baseline subtracted IV curve for R-baclofen (n = 6). Inset shows example currents at baseline (black) or in the presence of R-baclofen (dark grey). **c**) Baseline subtracted IV curves for R-isovaline at three concentration (50 μM n = 6; 250 μM n = 8; 1 mM n = 4). **d**) Example currents at baseline (black) and on application of three concentrations of R-isovaline (dark grey). **e**) Baseline subtracted IV curve for S-isovaline (n = 4). Inset shows example currents at baseline (black) or in the presence of S-isovaline (dark grey). **f**) Summary graph showing the currents recorded at -112.5 mV at baseline (open bars) or on application of GABA, R-baclofen, R-isovaline or S-isovaline (shaded bars). Data are represented as mean ± SEM. *** = p < 0.001.

We then transiently transfected AtT-20 cells with the GABA_B1a_ and GABA_B2_ subunits and GFP. To confirm membrane expression of GABA_B_ receptors in AtT-20 cells, we used a mouse monoclonal antibody to the GABA_B1_ subunit, which is unable to traffic to the cell surface without GABA_B2_ [4–6]. Fig. 2A,B illustrates cell surface expression of GABA_B1_ and colocalisation with GFP alongside the negative control. Next, we assessed if AtT-20 cells transiently transfected with both the GABA_B1a_ and GABA_B2_ subunits responded to GABA, R-baclofen or R- and S-isovaline. GABA (1 μM) applied for 2 min produced an inwardly rectifying current that was antagonised by co-application with 1 μM CGP 52432 (Fig. 3A). GABA evoked a maximum current of -41.0 ± 9.7 pA. Co-application of GABA and a GABA_B_ antagonist, CGP 52432, reduced the current to -8.0 ± 4.1 pA (paired t-test, t = 4.316, df = 6, p = 0.005, n = 7). The reversal potential of the GABA evoked current was -52 ± 3 mV (95% CI: -59 mV to -45 mV, n = 7).

As shown in Fig. 3B, R-baclofen (5 μM, 2 min) also induced an inwardly rectifying current. The maximum evoked current was -44.5 ± 16.5 pA. The mean reversal potential of the baclofen-evoked current was -44.0 ± 2.0 mV (95% CI: -48 to -40 mV, n = 6). These data, summarised in Fig. 3F, demonstrate that GABA and R-baclofen produce significant changes in current as determined by repeated measures 2-way ANOVA with Bonferroni’s post hoc test (interaction F_(5,29)_ = 5.48, p = 0.0011; GABA -118.5 ± 12.9 pA vs baseline -77.4 ± 8.8 pA, p < 0.001, n = 7; R-baclofen -101.4 ± 12.3 pA vs. baseline -56.9 ± 10.9 pA, p < 0.001, n = 6)

In contrast to GABA and R-baclofen, R-isovaline (5 min) did not produce a statistically significant change in the measured current (Fig. 3C,D,F). R-isovaline currents at 50 μM, a submaximal effective concentration in thalamocortical slices [17], were -56.4 ± 7.8 pA vs. a baseline of -62.5 ± 8.9 pA (p > 0.05, n = 6). Furthermore, the mean current after application of 250 μM R-isovaline, a ceiling concentration for imparting physiologic effect in thalamocortical slices [17], was -72.8 ± 8.3 pA vs a baseline of -67.5 ± 11.4 pA (p > 0.05, n = 8) and at 1 mM R-isovaline, they were -70.3 ± 28.8 pA vs. a baseline of -66.1 ± 23.0 pA (p > 0.05, n = 4). A high concentration of the S-enantiomer of isovaline (500 μM for 5 min, Fig. 3E,F) also did not produce a change from baseline current (-82.2 ± 4.8 pA vs baseline -89.2 ± 11.3 pA, p > 0.05, n = 4).

**Fig 4:**
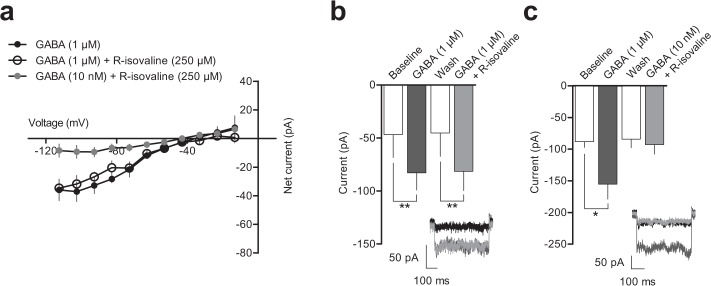
R-isovaline does not modulate GABA-evoked currents in transfected AtT20 cells. **a**) Baseline subtracted IV curves for GABA (1 μM; n = 3) and co-application of R-isovaline with GABA (1 μM n = 3; 10 nM n = 6). **b**) Graph shows that R-isovaline does not alter the current evoked by a high concentration of GABA. Inset shows example currents at baseline (black), on application of GABA (1 μM; dark grey) and on co-application of 1 μM GABA with 250 μM R-isovaline (light grey). **c**) Graph shows that R-isovaline does not alter the current when co-applied with a low concentration of GABA. Inset shows example currents at baseline (black), on application of 1 μM GABA (dark grey), and on co-application of 10 nM GABA with 250 μM R-isovaline (light grey). Data are expressed as mean ± SEM. * = p < 0.05, ** = p < 0.01.

To test if R-isovaline could modulate GABA action, we applied GABA (1 μM) in the presence or absence of R-isovaline (250 μM). As shown in Fig. 4A,B there was no statistically significant difference in the currents with application of GABA alone (-82.9 ± 16.3 pA) compared to GABA + R-isovaline (-81.5 ± 18.1 pA, repeated measures 1-way ANOVA with Bonferroni’s post hoc test, F_(3,2)_ = 24.41, p > 0.05, n = 3). To determine if R-isovaline is a positive modulator, GABA was applied at 1/100 of an effective concentration in the presence of R-isovaline after confirming a response to a high concentration of GABA. As shown in Fig. 4A,C, co-application of R-isovaline with a low concentration of GABA did not significantly change the measured current as determined by repeated measures 1-way ANOVA with Bonferroni’s post hoc test (GABA + R-isovaline -92.7 ± 15.0 pA vs. wash -84.3 ± 13.4 pA, F_(3,5)_ = 6.131, p > 0.05, n = 6). R-isovaline did not positively modulate or occlude GABA-induced inward currents.

**Fig 5:**
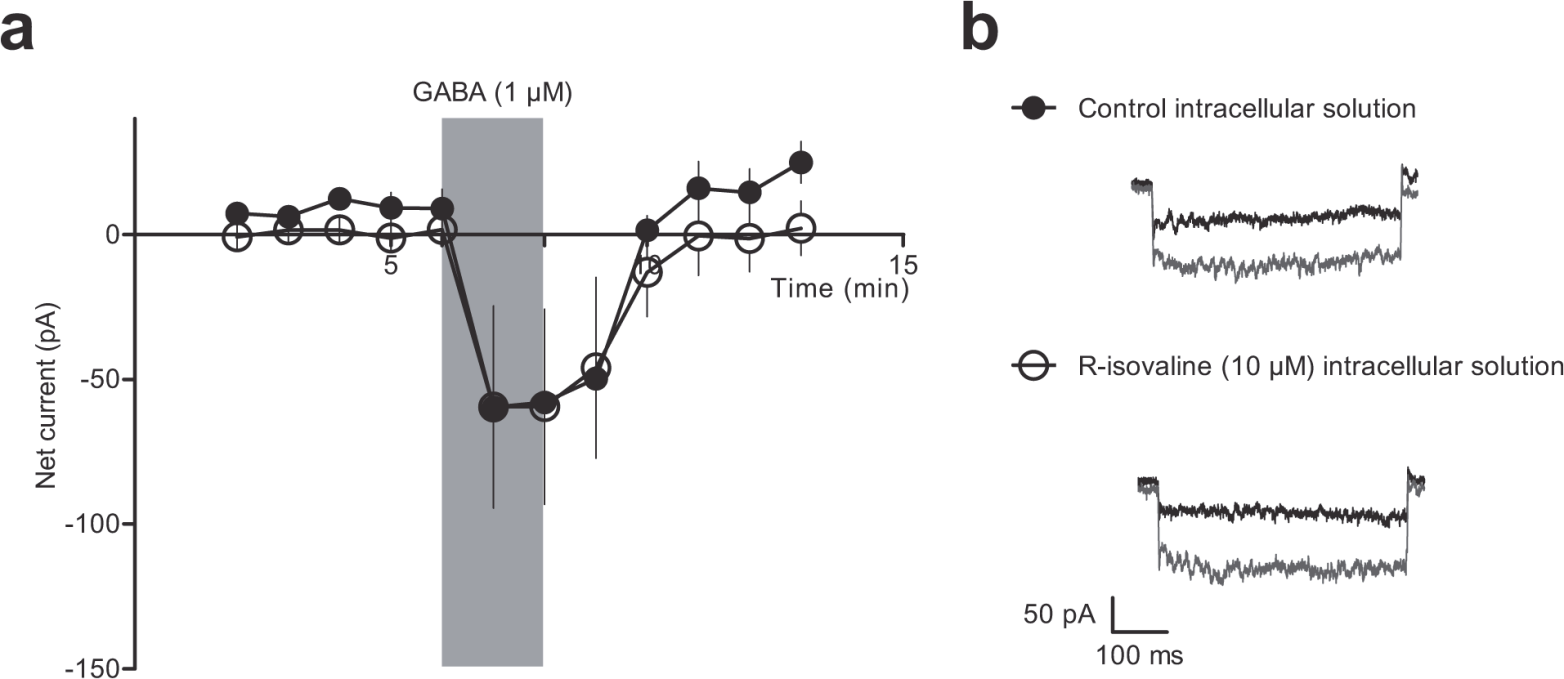
Intracellular R-isovaline does not alter baseline or GABA-mediated currents in transfected AtT-20 cells. **a**) Baseline subtracted current at -112.5 mV over time for control intracellular solution (n = 6) and intracellular solution containing R-isovaline (10 μM; n = 6). **b**) Example currents at baseline (black) or during 1 μM GABA application (dark grey) recorded with electrodes containing control solution (top) or intracellular solution containing R-isovaline (bottom). Data represent mean ± SEM.

To investigate the possibility that R-isovaline could only induce a current by an intracellular action, we applied R-isovaline via the intracellular pipette solution to AtT-20 cells while measuring the GABA-evoked inward current. We assumed that the concentration of R-isovaline that would transport across the cell membrane would be lower than that applied extracellularly. Therefore, we used a lower concentration for these experiments (10 μM). There was no difference in the currents recorded with electrodes containing R-isovaline or control solution as determined by a 2-way ANOVA (main effect of R-isovaline F_(1, 105)_ = 1.77, p = 0.2, n = 6 for both electrode groups, Fig. 5A,B). The difference in the GABA-evoked current recorded with electrodes containing R-isovaline or control solution was not statistically significantly different (control -59.5 ± 33.6 pA vs. R-isovaline -58.0 ± 17.7 pA, Bonferroni’s post hoc test, p>0.05).

**Fig 6:**
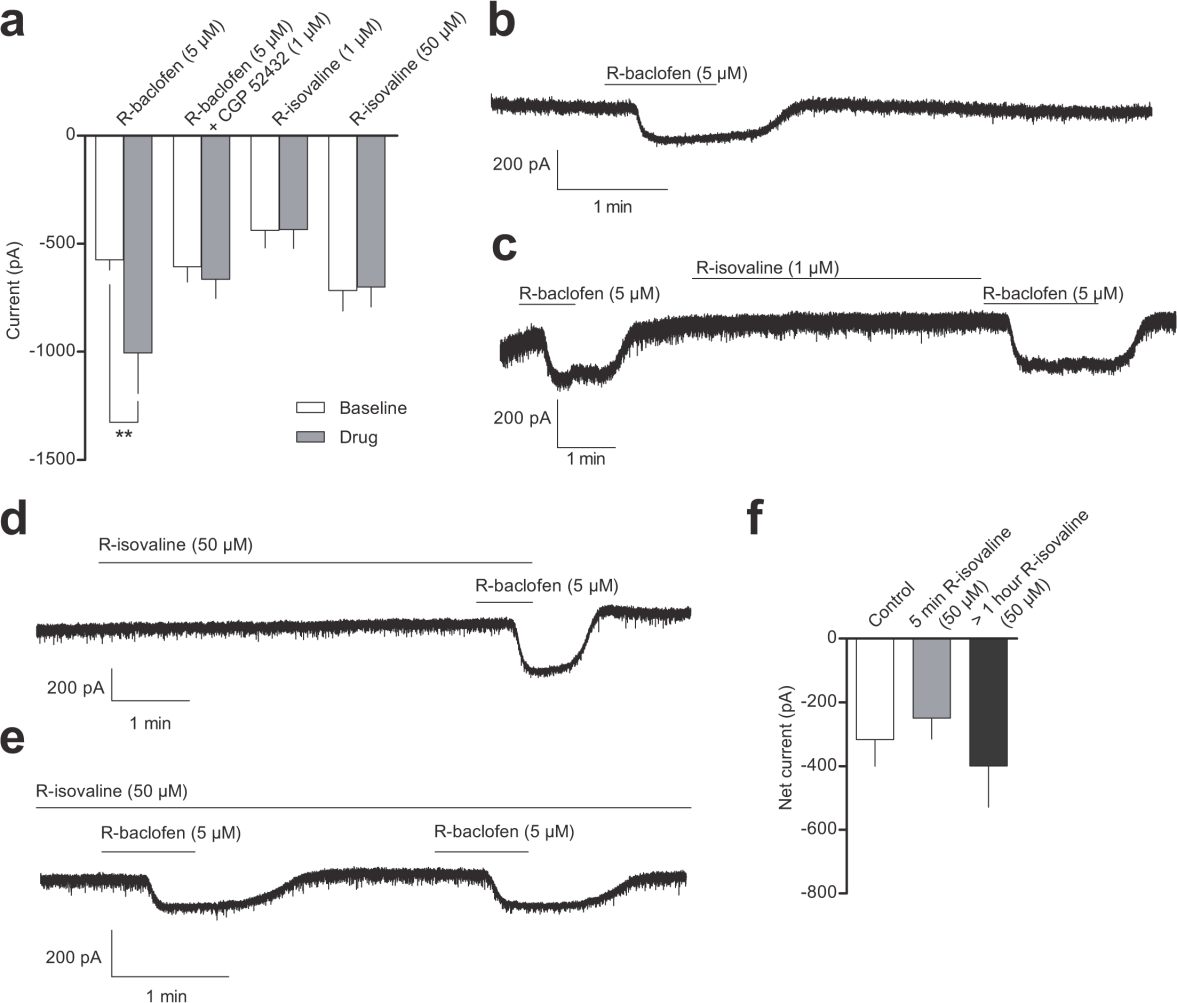
R-isovaline does not activate GABA_B_ receptors in cultured hippocampal neurons. **a**) Current at a holding potential of -80 mV in a high extracellular K^+^ solution at baseline (open bars) and on application of R-baclofen (n = 7), R-baclofen + CGP 52432 (n = 7) and R-isovaline at low (1 μM; n = 3) and high (50 μM; n = 8) concentrations (shaded bars). **b**) Example shows R-baclofen-mediated current **c**) Example shows lack of effect of low concentration of R-isovaline in a cell that responded to R-baclofen both before and after R-isovaline application. **d**) Example shows the effect of R-baclofen after 5 min pretreatment with 50 μM R-isovaline. **e**) Example shows the effects of two applications of R-baclofen after prolonged incubation (> 1 hour) with R-isovaline. **f**) Graph shows the magnitude of the current evoked by 5 μM R-baclofen in control extracellular solution (n = 8), after 5 minutes pretreatment with R-isovaline (n = 4) or after > 1 hour pretreatment with R-isovaline (n = 8). Data represent mean ± SEM. ** = P < 0.01.

One caveat with expression systems is that transfected receptor subunits may not associate with auxiliary proteins, scaffolding proteins or signal transduction pathways that exist in neurons that natively express GABA_B_ receptors. Therefore, we tested the effects of R-isovaline in cultured hippocampal neurons, examining membrane currents at a holding potential of -80 mV in a high K^+^ extracellular solution to enhance inward K^+^ currents. Cultured hippocampal neurons were chosen due to their well characterised response to GABA_B_ receptor agonists [21]. R-baclofen (5 μM) induced reversible inward currents in all neurons tested, determined to be significant by a repeated measures 2-way ANOVA (R-baclofen -1005 ± 187 pA vs. baseline -547 ± 48 pA, interaction F_(3, 21)_ = 4.01, p < 0.02, Bonferroni’s post hoc test, p < 0.01, n = 7, Fig. 6A,B). The currents evoked by R-baclofen were antagonised by 1 μM CGP 52432 (R-baclofen + CGP 52432 -665 ± 88 pA vs. baseline -607 ± 70 pA, p > 0.05, n = 7, Fig. 6A). R-isovaline (1 μM or 50 μM; 5 min [17]) did not produce an observable change in membrane current in this preparation (1 μM R-isovaline -434 ± 86 pA vs. baseline -438 ± 81 pA, p > 0.05, n = 3; 50 μM R-isovaline -701 ± 92 pA vs. baseline -716 ± 94 pA, p > 0.05, n = 8, Fig. 6A,C).

Finally, we assessed if R-isovaline could alter the response to R-baclofen in cultured hippocampal neurons. Application of 50 μM R-isovaline for 5 min (Fig. 6D,F) in high K^+^ extracellular solution prior to co-application with R-baclofen did not alter the R-baclofen evoked current compared to the current evoked by R-baclofen in control extracellular solution (control -316 ± 84 pA, n = 8; 5 min R-isovaline pretreatment -249 ± 66 pA, n = 4). To test if a longer incubation with R-isovaline was required, we incubated hippocampal cells with 50 μM R-isovaline for over 1 hour prior to and also during hippocampal current recordings. Prolonged incubation with R-isovaline also failed to affect the R-baclofen evoked current (> 1 hour R-isovaline -400 ± 130 pA, n = 8, Fig. 6E,F). Analysis with a 1-way ANOVA found no significant difference between any of the treatment groups (F_(2,17)_ = 0.40, p = 0.68).

## Discussion

Our studies show that R-isovaline did not induce an inward current in AtT-20 cells heterologously expressing GABA_B_ receptors or in cultured hippocampal neurons natively expressing GABA_B_ receptors. While GABA and baclofen activated GABA_B_ receptors, co-application with R-isovaline did not occlude or modulate GABA or baclofen responses. The data are in contrast to reports that show R-isovaline-mediated activation of GABA_B_ receptors [2,16]. However, others have demonstrated that isovaline does not alter the postsynaptic electrical properties of hippocampal pyramidal neurons [22].

Mouse pituitary AtT-20 cells contain endogenous GIRK channels [23], a common effector channel of GABA_B_ receptors [15]. In the present experiments, AtT-20 cells transiently transfected with GABA_B_ receptor subunits responded to GABA or R-baclofen with an inwardly rectifying current that reversed near the calculated equilibrium potential for K^+^. GABA_B_ receptors mediated the inward currents as confirmed by antagonism with CGP 52432. R-baclofen also induced a GABA_B_ receptor-dependent inward current in cultured hippocampal neurons, attributable to activation of GIRK channels [21].

In contrast to the GABA- or R-baclofen-induced activation of endogenously expressed GIRK channels, R-isovaline did not produce a response in either cell type. Previous work has demonstrated that R-isovaline induces a large increase in GABA_B_-mediated K^+^ conductance in thalamocortical neurons of brain slices, albeit with slower response kinetics than with R-baclofen [16,17]. One explanation for the difference may be that R-isovaline only acts at a specific isoform of the GABA_B_ receptor. GABA_B_ receptors are obligate heterodimers consisting of a GABA_B1_ and GABA_B2_ subunit [4–6,24]. While there is only one isoform of the GABA_B2_ subunit, two isoforms of the GABA_B1_ subunit are functionally expressed in the mammalian central nervous system [7]. If R-isovaline interacted selectively with GABA_B_ receptors containing the GABA_B1b_ subunit, we would not observe an effect because AtT-20 cells were transfected with cDNA coding only for the GABA_B1a_ subunit. However, GABA_B1b_ subunits differ only on the N terminus, a region that has been shown not to affect ligand binding or receptor function [9,25,26]. There are no known pharmacological or functional differences between GABA_B1a_- or GABA_B1b_- subunit containing GABA_B_ receptors upon heterologous expression, thus, a selective action at a specific isoform of the GABA_B1_ subunit is unprecedented [7,26].

Another explanation for the failure of isovaline to activate GABA_B_ receptors in our studies is that its action could depend on an alternative protein that associates with the GABA_B_ receptor or signalling components. GABA_B_ receptors can display pharmacological and functional differences depending on cell type and subcellular location. This is due to variation in cell specific proteins that regulate GABA_B_ receptors or their responses [27–34]. Cooke et al. (2012) [16] suggested a cell-specific mechanism of action in thalamic slices because significantly fewer neurons responded to R-isovaline than to R-baclofen; in addition, a subset of neurons responded to R-baclofen but not R-isovaline [16]. Candidate cell specific proteins include potassium channel tetramerization domain (KCTD) proteins, which directly associate with GABA_B_ receptors to alter ligand affinity and response kinetics such as desensitization [27]. However, differential expression of KCTD proteins may not explain a lack of effect of R-isovaline on K^+^ currents in hippocampal pyramidal neurons because the hippocampus has been reported to express all 3 KCTD proteins that act as GABA_B_ auxiliary subunits [27]. Alternatively, specific regulators of G-protein signalling proteins that alter GABA_B_ receptor efficacy [28,31] may be required for R-isovaline to have sufficient efficacy to evoke GABA_B_-mediated GIRK currents. It also is possible that isovaline acts as a biased agonist [35] at GABA_B_ receptors in such a way that it does not influence the membrane delimited coupling of the GABA_B_ receptor to GIRK channels. Instead, isovaline may initiate GABA_B_-mediated signalling cascades that indirectly influence membrane conductances, for example activation of Src-kinases [36,37].

Isovaline’s effects, particularly in the central nervous system, may depend on cellular location. For example, isovaline has anti-epileptic properties which are not likely GABA_B_-mediated, but instead are postulated to result from a selective enhancement of hippocampal interneuronal activity by non-synaptically increasing inhibitory input and/or eliciting a shunting phenomenon onto pyramidal neurons [22,38]. Our experiments demonstrated no effect of isovaline on hippocampal pyramidal neurons, but do not exclude isovaline actions on interneurons.

In summary, these studies demonstrate that R-isovaline does not activate GABA_B_-mediated GIRK currents in AtT-20 cells or isolated hippocampal pyramidal neurons. Furthermore, intracellular or extracellular application of R-isovaline does not occlude or modulate the actions of other GABA_B_ receptor agonists in these isolated cell systems. Future studies should be aimed at determining if cell specific modulators of GABA_B_ receptor signalling are required for isovaline-induced K^+^ currents in thalamic and other responsive neurons.
